# 24.3 EIGHTEEN-YEAR COURSE OF COGNITIVE FUNCTIONING IN PSYCHOTIC DISORDERS: FINDINGS FROM THE SUFFOLK COUNTY MENTAL HEALTH LONGITUDINAL STUDY

**DOI:** 10.1093/schbul/sby014.098

**Published:** 2018-04-01

**Authors:** Fett Anne-Kathrin, Eva Velthorst, Avi Reichenberg, Camilo Ruggero, Jennifer Callahan, Evelyn J Bromet, Roman Kotov

**Affiliations:** 1 City University of London; 2 Icahn School of Medicine at Mount Sinai; 3 University of North Texas; 4 Stony Brook University

## Abstract

**Background:**

Knowledge about the long-term cognitive course in psychotic disorders is limited. In this 18-year follow-up of participants of the Suffolk County project we report on the longitudinal course of cognitive performance in individuals with schizophrenia spectrum disorders, affective psychoses and other psychoses. We investigate (i) change in functioning in 6 cognitive domains from 2-years to 20-years follow-up after first admission; (ii) 20-year performance and age-related differences in cognitive performance in patients relative to a never psychotic comparison group; and (iii) key predictors of clinically meaningful cognitive decline in patients.

**Methods:**

Data came from the Suffolk County Mental Health Project, a prospective study of first-admission patients. Cognitive tests were administered 2 years (n = 399; schizophrenia spectrum: 285, affective psychoses: 226, other psychoses: 117) and 20 years (n = 240; 115, 92, and 34, respectively) after first admission, with 195 individuals completing cognitive tests at both time points. A never psychotic comparison group (N=260) was assessed at year 20.

**Results:**

Individuals with schizophrenia spectrum disorders showed lower cognitive functioning than those with affective and other psychoses. Over time, patients declined in cognitive performance on almost all tests (d = 0.24 (range 0.12- 0.44)) with comparable magnitude across diagnoses. Longer duration of untreated psychosis and low childhood IQ were significantly associated with clinically relevant decline (>0.5SD) in general verbal ability and processing speed, but there were no robust predictors of cognitive decline across tests. Cross-sectional comparisons of patients to controls showed increasing impairments with age for general verbal ability, verbal fluency, and executive functioning.

**Discussion:**

Our findings indicate that cognitive functioning in psychotic disorders continues to decline after the illness onset, that this decline is not specific to schizophrenia but present across psychotic disorders, and that, relative to never-psychotic individuals, impairments on some key-cognitive domains worsen with age. These results imply that cognitive treatment should not only include cognitive remediation but also prevention of age-related cognitive stagnation and/or decline.

